# Clinical decision making in postmastectomy radiotherapy in node negative breast cancer

**DOI:** 10.3332/ecancer.2018.874

**Published:** 2018-09-26

**Authors:** Rodrigo Barrientos, Suraj Samtani, Michael Frelinghuysen, Camilo Sotomayor, Juan Guillermo Gormaz, Mauricio Burotto

**Affiliations:** 1 Department of Radiation Oncology, Instituto de Radiomedicina IRAM, Santiago, Chile; 2 Radiation Oncology Unit, Hospital Clinico Regional de Concepción, Concepción, Chile; 3 Department of Medical Oncology, Clinica Alemana Santiago, Vitacura, Chile; 4 Centro de Investigación Clínica Bradfordhill, Santiago, Chile

**Keywords:** postmastectomy radiotherapy, node negative breast cancer, breast cancer radiotherapy

## Abstract

For decades, postmastectomy radiotherapy (PMRT) has been recommended for node positive [N(+)] breast cancer patients; nevertheless, the beneficial effect of PMRT for treatment of node negative [N(−)] disease remains under discussion. Nowadays, the biology of breast cancer and the risk factors (RFs) for locoregional failure (LRF) must be included in the decision on whether or not to carry out PMRT. For these reasons, the present review aims to evaluate the rationale use of PMRT in N(−) patients and discuss which subgroups may further benefit from the treatment in present times where the decision must be personalised, according to the RFs of locoregional recurrence (LRR). To perform the analysis, we ponder that LRR of over 10% should be considered unacceptable due to the fact that LRRs generate great morbidity in patients. For this purpose, we consider that routine RT in these patients is not recommended, although there are subgroups of patients with high LRR, in which PMRT could be beneficial.

## Introduction

Currently, breast cancer is the second most prevalent cancer in women, after nonmelanoma skin malignant diseases. In fact, it is estimated that 30% of new cancer cases and 25% of deaths caused by cancer in women are due to breast cancer in 2018 [1], with a cumulative incidence and mortality of 126.01/100,000 and 27.91/100,000, respectively [2, 3].

For decades, postmastectomy radiotherapy (PMRT) has been recommended for node positive [N(+)] breast cancer patients. Of note, between 1997 and 2005, three randomised controlled trials reignited the debate about the role of PMRT, especially in the specific clinical setting of N(+) disease patients [4–6]; however, the beneficial effects of PMRT for treatment of node negative [N(−)] disease remains under discussion [7].

The present review has the purpose of evaluating the indications of PMRT in N(−) patients and discusses which subgroups may further benefit from treatment in this setting. To analyse this issue, we ponder that locoregional recurrence (LRR) of over 10% should be considered unacceptable, based on node negative breast conservative randomised trials that showed LRR rates of 6.2%–6.7% [8, 9].

## Role of postmastectomy radiotherapy

A recent meta-analysis from the Early Breast Cancer Trialists’ Collaborative Group (EBCTCG) [7] investigated 22 trials that included 8,135 breast cancer patients who were treated with total mastectomy (TM), randomised to receive either further RT or observation. At 20 years of follow-up, N(+) disease patients who received PMRT showed a reduction in risk of LRR from 26% to 8.1%, with a reduction in absolute risk of breast cancer mortality of 8.1% (RR 0.84; 95% CI 0.76–0.94), whereas PMRT in N(−) disease patients increased overall mortality (RR 1.23; 95% CI 1.02–1.49, 2*p* = 0.03), despite the fact that current numbers show that mortality in N0 patients is low.

Locoregional relapse should always be considered for the analysis of PMRT effectiveness because radiation therapy exerts its main benefit decreasing its occurrence. In the context of mastectomised patients, LRR is associated with a diminished quality of life, higher risk of mortality and lack of satisfactory results after performing either surgical or radiation rescue therapy. To clarify the magnitude of this problem, once an LRR has been diagnosed, patients have decreased survival rates (ranging from 46% to 63%) [10–12] and higher risk of distant failure (DF) [13]. In fact, Locoregional control plays an important role in both overall survival (OS) and distant disease [10, 11, 13, 14]. The time of diagnosis is especially relevant considering that adjuvant chemotherapy (QT) has shown poor results and no OS benefit after isolated LRR [15–18].

For all these reasons, and the fact that rational use of irradiation according to normal tissue tolerance doses is rarely associated with acute skin moist desquamation and late adverse effects (such as rib fracture, radiation pneumonitis, ischaemic heart disease and second malignancies) [19–22], we think that certain subgroups of N0 patients would benefit from receiving PMRT.

## Why PMRT may benefit different subgroups of N(−) patients?

Breast cancer is a heterogeneous disease with several molecular subtypes. Perou *et al* classified breast cancer into four distinct molecular subtypes based on a genetic profile: Luminal A, Luminal B, Her2 enriched and basal-like [23, 24], each of them with variable prognosis and different survival rates. In fact, Triple Negative Breast Cancer (TNBC) (a surrogate of basal-like disease) is associated with an increased risk of LRR [25, 26] and breast cancer specific survival (BCSS) [27]. This continues to be a topic under active investigation, but a proper analysis of the data can help in taking a proper decision regarding whether a patient could benefit from PMRT.

As a matter of fact, RT seems to have a role in the adjuvant management of T1-3N0 patients who have received mastectomy when molecular subtypes are considered; however, these benefits are still not fully understood [28, 29]. Moreover, consideration of genetic profiles in clinical practice is associated with technical and economic limitations, especially in developing countries. The Onco*type*DX recurrence score can predict LRR in breast cancer patients [30]; however, there is currently no clear evidence that can assist in decision making for adjuvant RT. Thus, up to this date, clinical-pathologic biomarkers are still used as a surrogate for genetic profile.

Some proven clinical-pathological factors that influence local control in breast cancer are age, tumour size, lymphovascular invasion (LVI), histological grade and no use of systemic QT [31–35]. Therefore, since RT exerts benefits, particularly in LRR, one could expect that PMRT could play a role in patients who present these factors as they are at higher risk of LRR.

## Radiation therapy in pathologic stage T1-T2 N0 patients

There is no clear evidence that T1-2N0 breast cancer patients may benefit from PMRT. The incidence of LRR is low and many patients at risk of failure would need to be treated in order demonstrate a benefit.

Two independent data sets were pooled and analysed by Truong *et al* [28]. At a median follow-up of 4.3 years, they reported on 1994 mastectomised breast cancer patients, treated without adjuvant RT between 1998 and 2009, LRR rates of 1.8%, 3.1%, 1.7%, 1.9% and 1.9% in luminal A, luminal B, Her2 (+) luminal, Her2 (+) non-luminal and TNBC breast cancer patients, respectively. Multivariate analyses reported higher risk of LRR for tumours > 2 cm, lobular histology and presence of close or positive margins. Thus, these factors should be considered along with diverse molecular factors to ponder the risk of LRR after PMRT. Nevertheless, it is important to take into account that systemic therapy was less commonly used in TNBC patients compared to other subtypes of breast cancer (*p* < 0.001), which might have influenced the results.

In the same way, Abdulkarim *et al* [29] analysed 768 patients, T1-T3 N0-1 TNBC patients treated with breast conservative treatment (BCT), modified radical mastectomy (MRM) without RT and MRM plus RT with a median follow-up of 7.2 years. The multivariate analysis showed that both LVI and lymph node positivity (N+) were associated with an increased risk of LRR. In this population, 468 patients with T1–2N0 tumours with similar clinic pathological features were treated with BCT (*n* = 233) or MRM without RT (*n* = 235). Five-year LRR-free survival for T1-2N0 was 96% and 90% in the BCT and MRM groups, respectively (*p* = 0.022). The Cox multivariate analysis showed that MRM without RT was the only independent prognostic factor associated with increased risk of LRR in patients with TNBC, compared to BCT (HR 2.53; 95% CI 1.12–5.75, *p* = 0.02). Adjuvant QT was not significantly associated with decreased risk of LRR.

From a study that investigated 390 mastectomies TNBC patients, including 307 T1-2 N0-1 patients, Chen *et al* [36] reported an LRR rate of 7.9%. This study suggests that after radical mastectomy, TNBC tumours have low LRR and maybe other clinical and biological factors, besides receptor expression, may influence locoregional control.

In order to determine which subgroup of patients have a high risk of LRR, Emin Yildirim *et al* reported on 502 patients T1-2N0 treated with TM plus axillary dissection enrolled between 1990 and 2004 an LRR rate of 2.8%, at a median follow-up of 77 months. The authors observed an inverse relationship between disease free survival (DFS) and tumour size. They also found that LVI positive patients had 13.2% of LR, compared to 1.2% in LVI negative patients [37]. For the analysis, they created two groups of patients: patients < 40 years and patients > 40 years old. Considering this subgroup analysis, they established at 10 years, that younger patients (<40 years) with ≤ 1 RF (tumour size and LVI) had an LRR free survival (LRRFS) of 98%, compared to 44% in those patients with > 1 LRR RF (HR 2.2; 95% CI 1.2–3.2, *p <* 0.0001) (Table 1), and established too that patients older than 40 years with 0–2 factors had a 99% LRRFS rate with respect to 57% in patients with three RFs (LVI, histological grade and tumour size) at 10 years (HR 2.0; 95% CI 1.3–2.8, *p* < 0.0001). Again, these data suggest that several factors must be considered when these types of patients are evaluated [37]. Likewise, Abi-Raad *et al* [34] analysed 1,136 patients T1-2N0 who have undergone mastectomy at a median follow-up of 9 years. They reported an LRR of 3.5% with a cumulative incidence of 5.2%. In a multivariate analysis, factors associated with an increased risk of LRR were LVI (*p* = 0.002), positive margins (*p* < 0.001), tumour size > 2 cm (*p* = 0.011), age < 50 (*p* = 0.018) and no use of systemic therapy (*p* = 0.036). The 10-year cumulative incidence of LRR in absence of any of these factors was 2%, increasing to 3.3% with one RF, to 5.8% with two RFs and 19.7% (95% CI 12.2–28.6%, *p* < 0.01) when three or more RFs were present.

These data were consistent with the results found by Jwa *et al* [38]; they analysed 390 T1-2N0-1 patients of which 307 were N0 patients. The factors associated with higher LRR in N0 patients at multivariate analysis were no use of adjuvant QT (HR 10.2; 95% CI 1.2–88.5, *p* = 0.04) and age < 50 years (HR 11.4; 95% CI 2.4–55.4, *p* = 0.01).

Another study, which aimed to evaluate which subsets of patients have a higher risk of LRR to benefit from PMRT, was carried out by Trovo *et al* [39]. Based on 150 T1-2N0-1a breast cancer patients, who were treated with radical mastectomy without adjuvant RT, they reported in the univariate analysis that premenopausal status (*p* = 0.004), presence of LVI, estrogen receptor (ER) negative (*p* = 0.02) and pathologic grade 3 tumour (*p* = 0.002) were associated with higher risk of LRR. In patients with these factors, the rates of LRR were 1.2%, 10.3%, 24.1% and 75% if one, two, three or four of these factors were present, respectively (*p* < 0.001).

To identify a subgroup of patients at higher risk of LRR who could benefit from PMRT, Troung *et al* [40] analysed 1,505 women treated with MRM over 10 years between 1989 and 1999. A recursive partitioning analysis was used, and LRR rates over 20% were reported in patients who presented LVI plus high tumoural grade, and in patients with tumour size ≥ 2 cm plus high-grade histology. Age and size were also evaluated in a study involving 1,019 T1-2N0-1 patients when 753 were N(−). Sharma *et al* [41] reported an LRR rate of 2.3%, with 10 years LRR rate in node negative patients of 10.5%, for patients with age < 40; meanwhile patients > 40 years had LRR rates of 1% (*p* < 0.0001). Among the 18 patients ≤40 years with T2N0 disease, the 10-year LRR rates were 18.6%, with younger age as the only significant independent predictor of LRR (HR 2.14; 95% CI 1.28–3.56, *P* = 0.004).

As per meta-analysis, close and positive margins are considered RFs for LRR in noninflammatory breast cancer [42]. Hastings *et al* [43] investigated 1,235 (1,259 cases) T1N0 patients who did not receive PMRT. With a median follow-up of 8.5 years, they observed a 10-year Kaplan–Meier LRR of 3.2% for all patients. Of note, the most common site of LRR was the chest wall (68%). In the multivariate analysis, two factors were significantly associated with higher LRR. These were the margins ≤ 3 mm (HR 2.97; 95% CI 1.21–7.29, *p* = 0.02) and G3 (HR 3.97; 95% CI 1.94–8.14, *P* = 0.0002). They also found that by combining these factors the 10-year Kaplan–Meier risk was 25% compared to 2.7% without them (*p* < 0.0001).

Ki 67, a nuclear protein present in cycling cells, is used as an indicator of tumour proliferation [44, 45], which has now been associated with worse DFS and OS [46]. Although there is not much literature that supports Ki67 as a single factor associated with a higher LRR risk, only one study has evaluated it as an RF for LRR and the role of PMRT. Selz *et al* [47] analysed 699 postmastectomy pN0 patients treated between 2001 and 1998 (191 patients with PMRT and 508 without PMRT), in order to establish the role of PMRT in LRR at a median follow-up of 56 months; they found only a Ki67 > 20% was an independent factor for LRR (HR 4.18; 95% CI 1.11–15.77, *p <* 0.0215).

In NSABP trials, 14 and 20 patients received lumpectomy plus axillary node dissection or MRM as a surgical procedure. All lumpectomy treated patients were required per protocol to receive standard breast irradiation (without regional nodal irradiation). Chest wall irradiation after mastectomy was not allowed per protocol. Of the 505 patients in both trials that underwent mastectomy, the 10-year Kaplan–Meier LRR, categorised by 21 Gene Assay Recurrence Score (RS) were 2.3%, 4.7% and 16.8% for low, intermediate and high groups, respectively (log-rank test, *p* ≥ 0.001) [30].

More recent prospective trial data have suggested certain subsets of pN0 patients may benefit from PMRT, in fact randomised single trial published by Wan showed that patients who receive PMRT had higher DFS that patients who were not irradiated. The main problem with this study is that it did not evaluate LRR, making the data interpretation difficult [48].

## Radiation therapy in pathologic stage T3N0 patients

Several randomised trials and retrospectives studies have addressed this issue in T3N0 patients. The Danish Breast Cancer Collaborative Group trials [49] evaluated the role of PMRT in high-risk patients. Of all randomised patients, 135 premenopausal and 132 postmenopausal women were N0. Postmastectomy radiation decreased locoregional failure (LRF) rate from 17% to 3% and 23% to 6% in premenopausal and postmenopausal patients, respectively (*p* values were not reported) (Table 1). A posterior analysis showed a smaller number of distant metastasis in patients who received PMRT [49]. Of notice, in these studies, axillary lymph node dissection was considered suboptimal with a mean number of 7 lymph nodes removed. Thus, it is possible that some of the reported T3N0 patients could have presented lymph node metastasis if additional axillary lymph nodes had been removed. Since then, several retrospective and population based studies have intended to provide recommendations, but there is no solid prospective data to support PMRT as a standard for N0 tumours larger than 5 cm. Helinto *et al* [50] in 38 patients reported significant differences of LRR rates between both groups, favouring PMRT.

Floyd *et al* [51] reported retrospectively a series of 70 T3N0 breast cancer patients who received radical mastectomy and systemic therapy without RT between 1981 and 2002. At a median follow-up of 85 months, 5-year survival and LRF rates were 83% and 7.6%, respectively. Of note, LVI was associated with higher LRR by log rank test (*P* = 0.017) with actuarial local failure rate of 21% for patients with LVI versus 4% for patients without LVI (Table 1). In the multivariate analysis, LVI status was significantly associated with lower locoregional free survival (LRFS) (*p* = 0.038, HR = 6.6), OS (p = 0.002, HR 5.0) and DFS (*p* = 0.036, HR 4.1), respectively. Taghian *et al* [52] evaluated 313 T3N0 mastectomised patients, without PMRT, treated in NSABP node negative trials (NSABP B-13, B-14, B-19, B-20 and B-23). Overall, 10-year cumulative incidence of isolated LRR was 7.1%. LRF with DF was 10.0%, while DF alone was 23.6%. Multivariate analysis did not identify significant prognostic factors for LRR. The authors concluded that PMRT should not be routinely used for these patients. A criticism of this study was that it did not evaluate all negative features for LRR.

Goulart *et al* [53] investigated 100 T3N0 breast cancer patients; 44 (44%) patients received adjuvant PMRT. At a median follow-up of 10 years, there was no difference in cumulative LRR rates between both groups; as depicted by 8.9% and 2.3% LRR rates in the no-PMRT and PMRT groups, respectively (*P = 0.2*). To consider, better BCSS rates were reported for patients with tumours > 5 cm and margin positives cases, compared to the smaller tumours. This was attributed to the fact that a greater number of patients with larger tumours received PMRT when compared to smaller tumours (18% versus 54%; *p* = 0.001). In addition, a greater number of patients with larger tumours received hormonotherapy (57% versus 32%; *p* = 0.03).

From a single institution database, Mignano *et al* [54] retrospectively evaluated 101 T3N0 breast cancer patients treated with mastectomy without PMRT, enrolled from 1974 to 1994. At a median follow-up of 93 months, LRR was 11%. There was no significant difference in LRR between tumours smaller or larger than 7 cm (*p* = 0.07).

Two studies from the Surveillance, Epidemiology and End Results (SEER) program database found mixed results. McCammon *et al* [55] performed a retrospective population-based analysis which included 1,844 women undergoing mastectomy and axillary staging from 1988 to 2002, considering a maximum follow-up of 180 months. Only a 33.8% of the sample received PMRT. The authors reported no significant differences in CSS when compared PMRT versus no PMRT. However, patients who received PMRT presented an OS of 70.7%, whereas patients who did not receive PMRT had OS rates of 58.4% with a median follow-up of 10 years (HR, 0.69; 95% CI 0.55–0.85, *p* < 0.01).

The other SEER database study [56] was performed in 2,525 women who underwent MRM between 2000 and 2010; 42% of the cohort received PMRT. At a median follow-up of 56 months, PMRT was associated with both OS and CSS with an HR of 0.63(*p* < 0.001) and 0.77 (*p* = 0.045), respectively. These studies were not designed to evaluate LRR, but apparently PMRT would have had a protective role in terms of OS and CSS.

## Discussion

Several studies have attempted to describe correlations between molecular subtypes and outcomes for breast cancer disease control and survival. Traditionally, it has been described that Her2neu positive disease and TNBC have an increased risk of LRR compared to Luminal A subtype. In their study of 2,985 patients, Voduc *et al* [25], Kyndi *et al* [26], Chen *et al* [36] and Albert *et al* [57] found significant differences in LRR rates based on molecular subtypes. The investigators reported LRFS and regional relapse–free survival of 92% and 96% for Luminal A subtype; 86% and 88% in Luminal B; 80% and 88% in Her2 (+) Luminal; 83% and 88% in Her2 (+) no luminal, and finally, 80% and 88% in TNBC, respectively. Tumour size and histological grade were found to be independent prognostic factors for this outcome.

Similarly, a series from MD Anderson Cancer Centre [58] described LRR rates of 28% in TNBC. Independent RFs for LRR in mastectomised patients were tumour size, positive margin, grade, presence of LVI and the lack of anthracycline-based QT.

Zumsteg *et al* [59] in a single institutional study evaluated 642 patients with T1–2 N0 disease. TNBC patients who were treated with TM presented an LRR rate of 5.4% versus 4.2% that went to conservative therapy. This suggests study a low recurrence risk instead of aggressive biology. Despite TNBC [29, 36, 39, 57] are associated with higher LRR and worse outcomes, molecular biology should not be the only factor to take into consideration because by itself it might be not important enough.

LVI has also been associated with BCSS and LRR [60–62]. Lee *et al* [61] analysed 2,760 node negative breast cancer patients in order to evaluate BCSS. They observed that LVI was associated with increasing tumour size (*p* < 0.0001), higher histological grade (*p* < 0.0001), less favourable Nottingham Prognostic Index group (*p* < 0.0001) and younger age (*p* < 0.0001). Moreover, LVI was a significant prognostic factor in the multivariate analysis for BCSS in patients with and without adjuvant therapy. In the context of LRR, Truong *et al* [40] observed on 763 T1–2N0 patients, at a median follow up of 7 years, that LVI was an independent prognostic factor for LRR (RR 2.32; 95% CI 1.26–4.27, *p* < 0.007). In the same way, Boutrus *et al* [62] studied 1.275 N0 women (692 premenopausal and 583 postmenopausal) who underwent mastectomy followed by systemic therapy. They identified LVI (*p* = 0.23) and tumour size> 2 cm (*p* = 0.027) as RFs for LRR in premenopausal women, while LVI was the only significant factor associated with loco-regional failure in postmenopausal women.

Wallgren *et al* [63] reviewed the records of 1.257 T1-3N0-1 patients treated with BCT between 1980 and 2003. The aim of the study was to evaluate LVI as a predictor of nodal failure. At median follow-up of 8 years (ranged, 0.1–21 years), LVI was present in 17% of patients. In the multivariate analysis, tumour size, high grade and local failure were significant predictors of regional nodal failure (*p* = 0.049,* p* = 0.013,* p* = 0.0001, respectively), whereas LVI did not show a significant relationship with regional nodal failure. The presence of LVI in T2 and T3 population did not increase the risk of regional nodal failure (*p* = 0.15). These results suggest that LVI should not be used as a sole indicator for regional failure. This is reaffirmed by a recent study in which it is shown that LVI is not the only factor to be taken into consideration when categorising a patient as having a high risk of failure. Although this study did not exclusively analyse patients, N0 is applicable to this group and to all patients with breast cancer [64].

Tumour size is another important prognostic factor associated with LRR [13, 28, 34, 53]. As tumour size increases, so does the risk of nodal compromise, and chance to develop distant metastases and mortality as well. In 1989, Carter *et al* [65] postulated that tumour diameter and nodal status were independent but additive in-patient`s prognosis. They established a linear relationship between size and nodal compromise. Similarly, high-grade tumours have been correlated with increased risk of recurrence and worse prognosis [5, 31].

Ki67, a cell proliferation marker, has been described as a prognostic factor for LRR after neoadjuvant QT [66]. Synnestvedt *et al* [67] reported an inverse correlation between high Ki67 levels and DFS, DDFS and CSS.

Similarly, Kilickap *et al* [68] correlated high Ki67 levels with ER (−), HER2 new positive and higher tumour grade. The investigators also reported that high Ki67 levels were correlated with more failures and decreased survival. Nevertheless, up to date, Ki67 has not been validated as a marker able to predict recurrence and thus should be used in association with other factors based on clinical judgment [68].

Another important variable is pathology quality assurance. The BIG 2.04 SUPREMO phase III trial evaluated pathologic inclusion criteria entry for the trial RT postmastectomy for intermediate-risk breast cancer and suggests after central review that 19% of node negative patients were ineligible for trial. These data raise questions about whether clinical trials need to be powered to accommodate significant minorities of patients actually being ineligible, or should they reflect practice in the real world [69]?

It is true that there are reports in the specific group of young patients, that the main risk is distance failure, rather than LRR, it should be considered that breast cancer in patients < 40 years old, age is a RF for treatment failure (Table 1), independent of molecular subtype [70]. Azim *et al* analysed 1.360 patients with pT1–2 N0 tumours treated with BCT and found a local recurrence hazard ratio in women < 45 years old of 4.09 compared to women > 65 years old.

Younger patients tend to harbour more aggressive disease with higher rates of local failure and decreased survival. Therefore, we suggest that a multidisciplinary team should perform their evaluation and management.

## Conclusion

The decision to carry out PMRT to reduce the risk of LRF must be personalised. In this context, the use of PMRT in breast cancer patients with tumour size ≥ 5 cm must be recommended in the presence of one or more RFs such as young age (< 40 years or premenopausal status), no use of systemic treatment, large tumour size, margin status, LVI and molecular subtype due to the high risk of LRR. There is no evidence for PMRT in absence of these factors.

In the group of patients with T1–2N0 disease who have undergone mastectomy, we conclude that the decision of PMRT should be personalised and discussed, based on the knowledge of the disease, the clinical-pathological factors that affect it and the molecular factors that might influence the results, always in a multidisciplinary team with all those involved in the treatment of the patient. Also this treatment should be discussed with the patient, exposing the risks and benefits of the LRR and treatment. Despite the limitations of the studies analysed in this review and the fact that there is no prospective evidence to support this treatment, we considered the possibility of PMRT in patients with high risk of LRR.

## Conflicts of interest

None.

## Funding

The authors have no funding to report

## Abbreviations

BCSS: Breast cancer specific survival
BCT: Breast conservative treatment
CSS: Cancer specific survival
DF: Distant failure
DFS: Disease free survival
EBCTCG: Early Breast Cancer Trialists’ Collaborative Group
ER: Estrogen receptor
HT: Hormonal Therapy
LRF: Locoregional failure
LRFS: Locoregional free survival
LRNI: Locoregional lymph node irradiation
LRR: Locoregional recurrence
LVI: Lymphovascular invasion
MRM: Modified radical mastectomy
N(−): Node negative
N(+): Node positive
OS: Overall survival
PMRT: Postmastectomy radiotherapy
QT: Chemotherapy
RT: Radiotherapy
TM: Total mastectomy
TMX: Tamoxifen
TNBC: Triple negative breast cancer

## Figures and Tables

**Table 1.** T3 N0 PMRT.

Publication	No. of patients analysedT3N0	Median age	Follow-up	PMRT	LRNI	Systemic therapy	Global failure T3 N0	LRR in patients who received RT	Results	Multivariate and subgroup analysis
Maaret Helinto 1999	38	56 (35–84)	58 months (4.83 years)	In 33 patients	Four patients	Tamoxifen (TMX) → 37% CMF → 18%	5-year OS 72% DFS 77% LRR 18%	LRR with RT 9% LRR 60% without RT *P* = 0.003	RT better DFS (*p* = 0.04) RT better OS (*p* = 0.03)	No association found between factors
Scott R. Floyd 2006	70	50	85 months (7 years)	No	No	QT 41% TMX 24% Any systemic 56%	LRR 7.6% DFS 87%, 82% y 78% at 5, 10 and 15 years OS 82%, 72, 68% at 5, 10 y 15 years		LVI associated with lower OS (*p* = 0.0004), DFS (*p* = 0.023) and LRF (*p* = 0.017)	– LVI(+) HR 6.6 for LRF (*p* = 0.038) HR 5.0 for OS (*p* = 0.002) HR 4.1 for DFS (*p* = 0.036)
Alphonse G. Tanghian 2006	313	49 (29–74)		No	No	QT 34% TMX 21.1% QT+TMX 19.2%	Isolated LRF 8.9% Total LRF 10% Total DF 23%			No independent factors were identified for LRR
Jennifer Goulart 2011	100		PMRT7.5 years No PMRT 11.4 years	PMRT 44% No PMRT 56%	43% Chest wall y 57% Chest Wall and regional lymphnodes	PMRT – QT 50% – HT 64% No PMRT – QT 46% – HT 39%	LRR 6%	LRR – PMRT 8.9% – No PMRT 2.3% (*p* = 0.2) BCSS – PMRT 85.8% – No PMRT 74.6%		T3 > 5 cm better BCS HR 0.2; 95% CI 0.9*–0.6, *p* = 0.04) – Bigger tumours received more HT and PMRT Subgroup G3 presented 17% failure
Robert McCammon 2008	SEER (1998–2002) N° 1844 patients	No data	10 years	PMRT 33.8% No PMRT 66.2%	No data	No data	CSS inPMRT -- > HR 0.88 (*p* = 0.38) 10-year OS años – PMRT 70.7% – No PMRT 58.8% (*p* < 0.01)	No data		OS – HR PMRT 0.69 *p* < 0.01 – HR in > 50 years = 0.41, *p* < 0.01 Grade 1 HR = 0.6, *p* < 0.01
Matthew E. Johnson 2014	SEER (2000–10) N° 2525	No data	56 months (4.66 years)	PMRT 42.1% No PMRT 57.9%	No data	No data	OS a 96 meses – PMRT 76.5% – No PMRT 61.8% (p < 0.01) CSS 96 meses – PMRT 85% – No PMRT82% (p < 0.01)			PMRT had better OS – HR 0.63, p > 0.001 PMRT had better DSS – HR 0.77, p > 0.01 Histology grade I correlated with better OS and CSS
M Overgaard 82b 1997	135 patients T3N0	No data	114 months (9.5 years)	PMRT – 58 patients No PMRT – 77 patients	All with PMRT	135 patients with CMF	10-year DFS with PMRT 74% – No PMRT 62% 10-year OS – PMRT 82% versus No PMRT 70%	LRR – with PMRT 3% – No PMRT 17%		
M Overgaard1999	132 patients T3N0	No data	119 months(9.9 years)	PMRT – 68 patients No PMRT – 64 patients	All with PMRT	All received TMX	10-year DFS – PMRT 43% – No PMRT 40% 10-year OS – PMRT 56% – No PMRT 55%	LRRwith PMRT 6% – without PMRT 23%		
Yolanda D Tseng 2015	87 patientsT3N0	No data	50.1 months (4.17 años)	PMRT – 69 patients (79%) No PMRT – 18 patients (21%)	No data			No LRR		
Mignano, John 2007	101 patients		93 months (7.75 años)	No PMRT	No		LRR 11% without PMRT DF 11% without PMRT			

HT = Hormonal therapy; BCS= Breast cancer survival; LRNI= Locoregional lymph node irradiation

**Table 2.** T1–T2 N0 PMRT.

Publication	No. of patients	Included population	Follow-up	PMRT	Adjuvant treatment	LRR global failure	Outcomes
2005 Truong *et al*	1505	T1–2 N0 with TM	7 years	No	50.7%	7.8%	Recursive Partitioning Analysis
							10 year Kaplan–Meier LRR G3 versus G1–2 → 12.1% versus 5.5% (*p* < 0.0001)
							10 year Kaplan–Meier LRR G3+LVI versus G3 without LVI → 21.2% versus 9% (*p* = 0.0008)
							10 year Kaplan–Meier LRR for G3+T2+LVI (−) without systemic treatment versus G3+T2+LVI (−) with systemic therapy → 23.2% versus 9.2% (*p* < 0.001)
Yildrin 2007	502	T1–2 N0 with TM	77 months (6.4 years)	No	56.2%	2.8%	
							MV analysis in ≤ 40 years + > 2 cm → LRR HR 5.4 (*p* = 0.05)
							MV analysis in ≤ 40 years + LVI → LRR HR 9.0 (*p* = 0.004)
							MV analysis in > 40 years + > 3 cm → LRR HR 8.6 (*p* = 0.05)
							MV analysis in > 40 years + G3 → LRR HR 7.0 (*p* = 0.05)
							MV analysis in > 40 years + LVI → LRR HR 18 (*p* = 0.007)
							Estimated 10 year LRFS rates: 98% in ≤ 40 years old with 0–2 RFs (low risk) versus 44% with > 2 RF (high risk) (*p* < 0.0001)
							Estimated 10 year LRFS rates: 99% in < 3 RF (low risk) in >40 years old versus 57% in patients > 3 RFs (high risk) (*p* < 0.0001)
Trovo 2012	159 → 54% N0	T1–2 N0–1 with TM	75 months (6.25 years)	PMRT in N1	95%	11%	5 year Kaplan–Meier LRR in Patients LVI (+) versus LVI (−) → 19.1% versus 3.2% (*p* = 0.002)
							Kaplan–Meier LRR in Premenopausal versus postmenopausal status → 13.4% versus 4.8% (*p* = 0.004)
							Kaplan–Meier LRR inER (−) versus ER (+) → 25.8% versus 4.7% (*p* = 0.002)
							Kaplan–Meier LRR in G3 versus G1–2 → 16.4% versus 1.4% (*p* = 0.002) Kaplan–Meier LRR in patients with non or one, two, three or four LRR factors → 1.2%, 10.3%, 24.1% and 75% (*p* < 0.001)
Rita Abi-Raad 2011	1136	T1–2N0 with TM	9 years	No	61.6%	5.2%	LRR MV analysis in patients with Systemic treatment → HR 0.5 (*p* = 0.036)
							LRR MV analysis in patients with margins < 2 mm → HR3.3 (p = 0.001)
							LRR MV analysis in patients with Size ≥ 2 cm → HR 2.0 (*p* = 0.011)
							LRR MV analysis in patients with > 50 years old → HR 0.5 (*p* = 0.018)
							LRR MV analysis in patients with ILV (+) → HR 2.7 (*p* = 0.002)
							10 year LRR Kaplan–Meier without RFs 2%; LRR with 1 RF 3.3%; LRR with 2 RF 5.8%; ≥ 3 RF, 19.7% (*p* < 0.0001)
Xing Xing Chen 2013	390 → 307 N0 (78.7%)	TNBC T1–2 N0–1 with TM	60.5 months (5 years)	No	86%	7.9% global	RR MV analysis in patients < 50 years versus ≥ 50 years → HR 4.82 (*p* = 0.015)
							LRR MV analysis in patients with N(+) 3 versus 0–2 → HR 8.76 (*p* = 0.03)
							LRR MV analysis in patients with LVI (+) versus LVI (−) → HR 26.05 (*p* = 0.000)
							LRR MV analysis in patients with G3 tumour versus G1–2 → HR 2.87 (*p* = 0.039)
							5 year KM LRR with 0–1 RF was 4.2%; with 2 RF 25.2%; with > 3 RF 81% (*p* < 0.001)
Ranjna Sharma 2010	1019 → 753 N0	T1–2N0–1 with TM	7.47 years	No	76.9%	2.3%	10 year Kaplan–Meier LRR in N0 patients ≤ 40 years versus >40 years → 10.5% versus 1% (*p* < 0.0001)
							LRR MV analisys in N0 patients ≤ 40 years → HR 2.14 (*p* = 0.004)
							10 year Kaplan–Meier LRR in T1N0 and T2N0 in ≤ 40 years old patients was 9.3% and 18.6% (*p* not reported)
Troung 2014	1994 pacientes	T1–2 N0 with TM	4.3 years	No	80.5%		LRR MV analisys Lobular histology → HR 3.48 (*p* = 0.003)
							LRR MV analisys Close or positive margins → HR 3.42 (*p* = 0.009)
							LRR MV analisys Tumour size > 2 cm → HR 2.57 (*p* = 0.012)
Jessica Selz 2012	699 patients	T1–2 N0 with TM	56 months (4.6 years)	– 191 PMRT with RLI – 508 without PMRT	HT 65.5% CT 33.5% Trastuzumab 3.7%	Freedom from LRR: 97% patients	– LRR MV analisys in patients with with Ki67 > 20% → HR 4.18 (*p* < 0.0215)
							5 year LRRFS in PMRT versus no PMRT patients → 97.7% versus 96.8% (*p* = 0.663)
Bassam S. Abdulkarim 2011	768 TNBC – BCT+RT in T1–2 N0 → 233 – T1–2N0 MT with no PMRT→ 235	T1–2N0 with TM	7.2 years	No	69.9% in those with TM	LRR 10%	LRR MV analisys for MRM without RT versus BCT+RT in T1–2 N0 → HR 3.44 (p < 0.001)
							LRR MV analisys for MRM with RT versus BCT+RT in T1–2 N0 → 0.72 (*p* = 0.34)
Joseph Hastings 2014	1259 patients	TNBC T1 N0 with TM	8.5 years	No	QT 24.2%	10 year LRR 3.2%	LRR MV analysis in patients with margins ≤ 3 mm → HR 2.97 (*p* = 0.02)
							LRR MV analysis in patients in patien with G3 versus G1–2 tumours → HR 3.97 (*p* = 0.0002)
							10 year Kaplan–Meier LRR in Patients with one of these RFs versus two RFs → 2.7% versus 25% (*p* < 0.0001)

G = histological grade; BCT = breast conservative treatment; NS = not significant; RLI = regional lymph node irradiation
